# Understanding egorrhea from cultural-clinical psychology

**DOI:** 10.3389/fpsyg.2013.00894

**Published:** 2013-11-28

**Authors:** Jun Sasaki, Kaori Wada, Yoshihiko Tanno

**Affiliations:** ^1^Department of Clinical Psychology, Graduate School of Human Sciences, Osaka UniversityOsaka, Japan; ^2^Department of Educational and Counselling Psychology, McGill UniversityMontreal, QC, Canada; ^3^Department of Cognitive and Behavioral Science, Graduate School of Arts and Sciences, University of TokyoTokyo, Japan

**Keywords:** egorrhea symptoms, cognitive model, taijin-kyofusho, schizophrenia, social anxiety disorder, culture

## Abstract

Based on his observations in Japanese clinical settings, Fujinawa (1972) conceptualized egorrhea syndrome, which includes symptoms such as erythrophobia, fear of eye-to-eye confrontation, olfactory reference syndrome, delusions of soliloquy, delusions of sleep talking, and thought broadcasting. The key feature of this syndrome is self-leakage, a perceived sense that one's personal internal information, such as feelings and thoughts, are leaking out. To reach a more comprehensive understanding of egorrhea, this paper aims to present general overview and reconsider the phenomenon of self-leakage using cultural-clinical psychology as a framework. First, the symptoms of egorrhea are reviewed in relation to other related psychopathologies such as social anxiety disorder (SAD) and taijin kyofusho (TKS), as well as schizophrenia. Second, a series of empirical studies conducted using Japanese non-clinical samples are summarized. The results of these studies form the basis for subsequent discussions, which incorporates the cultural-clinical psychology perspective proposed by Ryder et al. (2011). This paper ends with a general discussion regarding implications for research and clinical practice.

## Introduction

The patient started experiencing a loss of interest and feelings of alienation around the age of thirteen. Around the same time, he began masturbating, which contributed to his sense of guilt and fear of what others might think of him. Increasingly, he became extremely anxious and self-conscious in social situations, to the point where he spent a few years hardly interacting with others at school.

At the age of 14, the patient became particularly concerned with his gas and believed his body was constantly giving off unbearable smells. He was convinced that others around him were disgusted by the bad smells he emitted and were badmouthing him. Eventually, he stopped going to school and spent much of his time in his room.

He was so convinced that the foul odor was caused by an abnormality with his anus, he decided to undergo a surgical treatment before starting high school. After the surgery, he was able to attend school, but remained overly self-conscious and unable to have conversations with his peers. However, his preoccupation with the foul odor soon returned; as a result, he began to once again isolate himself, and ultimately stopped attending school altogether. Furthermore, he developed a delusion that he was unconsciously broadcasting his thoughts—a conviction that he was talking to himself out loud in public without knowing it. He was paralyzed with the idea that he must be making inappropriate remarks in public, for example concerning his sexual fantasies.

Eventually, he was admitted to a hospital at the age of 17, where he continued isolating himself from other patients. By the time the case description was written, the patient had been hospitalized for seven years and his symptoms had never completely lifted.

The above vignette is a summary of a clinical case originally described in Fujinawa and Kasahara (1972). Within this and other patients, the Japanese psychiatrist Akira Fujinawa (1972) observed a cluster of symptoms characterized by a delusional belief that internal information, such as feelings and thoughts, are ‘leaking out’, and consequently put forth a conceptualization of “egorrhea symptoms.”

Egorrhea has not been listed in successive editions of the Diagnostic and Statistical Manual of Mental Disorders (DSM; American Psychiatric Association) and therefore remains little known to mental health professionals in Western countries. By contrast, the concept of egorrhea is a familiar term to mental health professionals in Japan, as it appears in the Japanese literature not infrequently (e.g., Tanaka, 1995; Masuda et al., 1998). In particular, the first and third authors have conducted empirical studies on egorrhea symptoms based on a cognitive-behavioral model (e.g., Sasaki and Tanno, 2003, 2004, 2005a,b,c, 2006; Sasaki, 2011).

The purpose of this paper is to provide an overview of egorrhea and reconsider it from the cultural-clinical psychology perspective. First, in order to familiarize the readers with the nature of egorrhea, the authors will describe the symptoms of egorrhea in relation to other related psychopathologies such as social anxiety disorder (SAD) and taijin kyofusho (TKS), as well as schizophrenia. Second, a series of empirical studies conducted by the authors using Japanese non-clinical samples will be summarized. The results of these studies will then be discussed in light of the cultural-clinical psychology perspective proposed by Ryder et al. (2011). Lastly, a general discussion pointing to implications for research and clinical practice will be provided.

## Nature of egorrhea

What is egorrhea? How is it similar or dissimilar to other psychopathologies? Since egorrhea remains relatively unknown to Western psychology, we begin this section by providing a brief explanation of each symptom and reviewing relevant research findings. In addition, features of egorrhea will be compared and contrasted with related psychopathologies, and gaps in the current literature will be identified.

### Key symptoms

Fujinawa (1972) delineated six symptoms of egorrhea: (a) erythrophobia (fear of blushing), (b) fear of eye-to-eye confrontation (*jiko-shisen-kyofu*), (c) olfactory reference syndrome (ORS), (d) delusions of soliloquy, (e) delusions of sleep talking, and (f) thought broadcasting.

Erythrophobia refers to fear of blushing in public (Uchinuma, 1990). Individuals with erythrophobia fear that others will notice they are embarrassed because of their blushing. Some people are more prone to blush than others, which may contribute to the onset and maintenance of erythrophobia. Although blushing is a common emotional response, many people consider blushing to be highly undesirable (Shields et al., 1990). Consequently, some individuals experience a great deal of distress due to blushing and develop a blushing phobia and subsequently seek treatment (Scholing and Emmelkamp, 1993; Mulkens et al., 1999).

The Japanese word for fear of eye-to-eye confrontation, jikoshisen kyofu, literally means “the fear of one's own gaze.” Individuals who suffer from this phobia imagine their gaze to be unusually sharp and ugly (Kasahara, 1972). Iwata et al. (2011) reported that patients with this condition are convinced that others are displeased and offended by their glare. Furthermore, Yasumatsu (1993) showed that fear of eye-to-eye confrontation was associated with depressive mood, fear of being looked at, and the feeling of being excluded or ridiculed by others.

Individuals with ORS are overly concerned with odors they believe their bodies emit, such as body odor, bad breath, urine and gas (Kasahara, 1972). A systematic interviewing of 20 patients with ORS showed that 85% of patients had delusional beliefs, 32% had attempted suicide, and 44% had sought non-psychiatric medical, surgical, or dental treatment for the perceived odor (Phillips and Menard, 2011).

Individuals with a delusion of soliloquy believe that they talk to themselves in public without being aware of it. By contrast, individuals with a fear of sleep talking are concerned that they will verbalize their secrets while asleep, and that other people are listening to them (Kasahara, 1972). In both cases, the sufferers think that they have no control over their utterances, and that their secret thoughts are heard by others. Unfortunately, little empirical research has been conducted on these conditions.

Lastly, thought broadcasting refers to a type of delusion that one's thoughts are transmitted to others (Miyamoto, 1973). It is one of Schneider's first rank symptoms of schizophrenia (Schneider, 1959), among other symptoms such as voices discussing or commenting on one's behavior, delusional perception, and thought withdrawal. First rank symptoms have been considered to be robust markers for schizophrenia diagnosis and prognosis, although more recently Thorup et al. (2007) questioned their predictive value in several outcome measures.

Egorrhea syndrome represents a psychological concept related to above six symptoms. What is common to all six symptoms of this syndrome is the delusional conviction of “leaking out,” whether it be blushing, body odor, sight, sleep talking soliloquy, or thoughts or feelings. Aside from these examples, individuals with egorrhea may also feel that they are transmitting a virus to other people (Sekine, 1986), or produce bodily noises conceived to be offensive (e.g., swallowing, stomach ramblings, and heart beats) (Kasahara et al., 1985). Furthermore, it has been documented that patients, especially those with fear of eye-to-eye confrontation or ORS, believe that others dislike them because whatever leaks out of their bodies is offensive to them (e.g., Fujinawa, 1972). However, it is said that prognosis of egorrhea patients is not that bad despite their delusional symptoms, as reviewed below.

Manifestations of symptoms vary from one patient to another. For example, one person may only have erythrophobia, while another may only exhibit thought broadcasting. In addition, according to Fujinawa (1972), egorrhea sufferers may also go through *symptom shift* (*shoujou-hensen*). That is, a patient who initially exhibits, for example, erythrophobia, can subsequently develop fear of eye-to-eye confrontation, ORS, a delusion of soliloquy, or thought broadcasting, as symptoms become more severe and daily functioning diminishes. Hagiuda and Hamada (1991) reported two cases of such symptoms shift. One patient initially presented with ORS, which shifted into thought broadcasting. In the other case, the individual first exhibited a delusional fear of facial twitching (*hyoujou-kyofu)*, which then shifted into fear of eye-to-eye confrontation and thought broadcasting. This suggests an underlying common mechanism across egorrhea symptoms.

### Egorrhea and related psychopathologies

How are egorrhea symptoms diagnosed? A closer look at the six symptoms reveals that the first three symptoms (fear of blushing, fear of eye-to-eye confrontation, and ORS) are related to social phobia and thus have been conceptualized as part of taijin-kyofusho (TKS). On the other hand, the delusional aspect is more prominent in the other three symptoms (delusion of soliloquy, delusion of sleep talking, and thought broadcasting), which have been conceptualized as being related to schizophrenia. The coexistence of social phobia and delusional aspects in egorrhea, coupled with observations of symptom shifts, suggests an important implication. That is, taijin-kyofusho (TKS) and schizophrenia may exist on a spectrum (e.g., Fujinawa, 1972; Kasahara, 1972; Yamashita, 1982), and symptom shifts may represent a progression of the syndrome from symptoms related to social phobia to those related to schizophrenia. Given this implication, the following section presents a review of pertinent literature.

#### Social anxiety disorder and TKS

The Japanese psychiatrist Masatake Morita conceptualized taijin-kyofu sho (TKS) as a form of social anxiety in the 1920s. TKS, which literally means the fear (*kyofu*) of interpersonal relations (*taijin*), is characterized by an intense fear that the individual displeases or embarrasses others as a result of their somatic characteristics, such as body order, facial expressions, or appearance. In previous DSMs, TKS was listed as a culture-bound syndrome specific to Japan. In DSM-5, TKS still remained in the Glossary of Cultural Concepts of Distress, although the updated description indicates that TKS is observed in other hierarchical societies such as Korea.

Because SAD and TKS share social fears and accompanying avoidant behaviors, there is confusion as to whether, and if so how, these two concepts overlap and differ from one another. The theoretical difference is that negative evaluation by others which is humiliating to *oneself* is central to SAD, while the primary concern of TKS suffers is how they make *others* uncomfortable (Kirmayer, 1991). Furthermore, cognitive-behavioral therapy (CBT) was found to be less effective for the symptoms of TKS than those of SAD (e.g., Kleinknecht and Dinnel, 2001; Rector et al., 2006). This result provides empirical evidence that TKS and SAD are distinctive, while suggesting that current CBT approaches do not address all aspects of TKS.

Kasahara (1972) classified TKS into four subtypes based on the severity of symptoms: (a) a transient type, which is usually found in adolescents or associated with life changes, (b) a neurotic type, which is related to nervous temperament (shinkeishitsu), (c) an offensive type, which involves delusions or ideas of reference to offend others, and (d) a secondary type, which is a phobic disorder associated with schizophrenia. In this classification, the first and second types are common across cultures and correspond to SAD as defined in the DSM, where egorrhea symptoms of ORS and fear of eye-to-eye confrontation correspond to the third type (i.e., offensive type) of TKS (Kasahara, 1972). Although the offensive type has traditionally been considered specific to Eastern populations (e.g., Takahashi, 1989; Kirmayer, 1991), recent studies have revealed the presence of the offensive type within university student samples (Kleinknecht et al., 1997; Dinnel et al., 2002) as well as in clinical samples (McNally et al., 1990; Clarvit et al., 1996) in Western countries.

In their efforts to clarify the difference between SAD and TKS, Kinoshita et al. (2008) proposed a conceptual model that divides SAD into subtypes. According this model, SAD is first divided based on whether or not offensive symptoms are present. SAD with offensive type is then further divided based on whether or not there is conviction of offensiveness. The latest edition of the DSM-5 (2013) incorporated the offensive symptoms as one of the symptoms in SAD. Furthermore, the DSM-5 included the fear of showing anxiety symptoms that might be negatively evaluated by others as a criterion for SAD. This fear of showing anxiety resembles the features of egorrhea; it involves one's concern that internal information, such as anxiety and tension, is discerned by others. Thus, egorrhea is not a rare, peculiar phenomenon, but one that has potential for advancing our understanding of social anxiety.

Until the latest publication of the DSM-5, previous DSMs treated TKS as a culturally distinctive disorder in Japan. As a collectivistic culture, Japanese society values group harmony, which demands that individuals maintain heightened sensitivity to others. Furthermore, as opposed to people in Western countries who tend to have an independent self-construal, Japanese peoples' self-perceptions are organized around an interdependent self-construal, which focuses on connectedness with one's surroundings (Markus and Kitayama, 1991). Thus, behaviors that promote one's internal attributes (e.g., personal desires and motives) have negative repercussions for interpersonal relations, especially when these behaviors disregard others' feelings and disturb the group's atmosphere (Hamaguchi, 1985; Heine et al., 1992). True feelings are often not directly expressed as the typical Japanese style of communication involves the notion of *tatemae (*façade, appearances) and *honne* (true feelings) (Doi, 1986). As a result, being successful in the domain of interpersonal relationships requires an ability to infer others' feelings while simultaneously hide one's own feelings appropriately.

Despite the value placed on group harmony, it is a mistake to assume that Japanese people have no trouble relinquishing their need to establish a sense of self and express their individuality. On the contrary, Japanese scholars have long theorized that it is this constant struggle to balance these conflicting demands that gives rise to the onset of TKS. For example, Kondo (1970) theorized that TKS results from the tension between opposite psychological needs: *hairyoteki-yosei*, which refers to the need to care for others and be liked by others; and *jikoshuchoteki-yosei*, which refers to the need to assert oneself and be superior to others. Likewise, Kawai (1975), who considered TKS from an ethical perspective, made a similar argument that TKS results from the conflict between the ethics of individuals (i.e., individual development and the establishment of sense of self) and the ethics of place (i.e., the maintenance of a state of social equilibrium). Indeed, studies on social anxiety and self-construal have shown a positive association between social anxiety and an interdependent self-construal, as opposed to a negative association between social anxiety and an independent self-construal (Dinnel et al., 2002; Moscovitch et al., 2005; Levinson et al., 2011).

#### Schizophrenia

Similar to schizophrenia, egorrhea symptoms encompass a delusional feature, such as delusion of reference. Delusions of reference are characterized by a false sense that one is referred or implicated in neutral events in one's immediate environment (Wong et al., 2012). For example, if a stranger sits next to an individual with delusions of reference on a train and coughs, this individual may believe that the stranger is evaluating him critically (when in fact the person coughed because she had a sore throat). Accordingly, it can also be said that individuals with delusions of reference hold a high degree of conviction (i.e., the extent to which individuals believe their experience to be true, cf. Peters et al., 1999). In case of egorrhea, the sufferers are convinced that their internal information which they believed to be leaking out is offending others, and that therefore they are disliked by others.

Furthermore, some of the egorrhea symptoms are similar to Schneider's first rank symptoms (Schneider, 1959) such as thought broadcasting, or derangement of ego. As a result, clinicians tend to assume that patients with egorrhea symptoms suffer from severe mental disturbance. However, it is not necessarily accurate to diagnose all individuals with egorrhea symptoms as having a delusion disorder or schizophrenia. Despite displaying delusion-like cognitions, people who present with egorrhea symptoms retain insight into their beliefs, unlike individuals who present with delusional disorders or schizophrenia. Moreover, it has been documented that in contrast to patients suffering from schizophrenia, patients with egorrhea symptoms rarely experience a collapse of personality, and can follow their daily routine, albeit imperfectly (e. g., Kasahara, 1972).

The difference between egorrhea and schizophrenia is an interesting topic that warrants further study. In the past decade, within the literature attention to non-schizophrenic psychoses has increased. For example, the special issue of the *Journal of Psychiatric Research* (2004) addressed the various types of psychosis in non-schizophrenic disorders, such as mood disorders (Tsuang et al., 2004), body dysmorphic disorders (Phillips, 2004), bipolar disorders (Ketter et al., 2004), and Alzheimer's disease (Schneider and Dagerman, 2004). Yet, treatment strategies have not been examined in these studies, largely because psychological mechanisms of non-schizophrenic psychoses remain unclear. Since egorrhea symptoms are also a type of non-schizophrenic psychosis, empirical studies on egorrhea symptoms may shed light on mechanisms and treatment options for other non-schizophrenic psychoses.

### Gaps in the literature

Previous literature on egorrhea symptoms has several limitations. First, it has been limited to case studies and theoretical papers that describe symptomatology. There is a dearth of empirical studies, particularly studies that identify factors affecting the onset and maintenance of egorrhea. In addition, little effort has been made to develop a testable model that explains how other variables, such as the strength of conviction and ideas of reference, contribute to the mechanisms of egorrhea. Second, the existent literature is based on clinical patients whose problems are severe enough for them to seek treatment from mental health professionals. Consequently, there is no epidemiological data that indicate how prevalent egorrhea symptoms are within non-clinical populations. The exclusive focus on clinical populations also perpetuates the assumption that egorrhea exists only among severely disturbed patients.

Lastly, careful attention should be paid to the influence of sociocultural factors on the etiology and maintenance of egorrhea syndrome. In the Western nosological systems of psychopathologies such as the DSM, peculiar, and exotic psychiatric disorders from other cultures, that seem unfamiliar to the West, are regarded as culture-bound syndromes (Tseng, 2006). However, consideration of cultural factors may reveal that a particular indigenous disorder is in fact an extreme form of cultural adjustment. At the same time, overemphasis on cultural explanations can be problematic—-as is the case with TKS, symptoms that are originally thought to be specific to a particular culture may in fact be present in other cultures (e.g., McNally et al., 1990; Clarvit et al., 1996). Once a group of symptoms are classified as a culture-bound syndrome, however, many efforts are made to research and theorize about cultural factors while overlooking other factors. Thus, paying attention to cultural and individual factors in a balanced way is essential to gaining an accurate understanding of egorrhea syndrome and to developing effective treatments for this syndrome.

## Cultural-clinical psychology

Given these gaps in the literature, it is evident that further advancement in egorrehea research should take cultural factors into consideration while aiming to elucidate factors that contribute to symptomatology. To this end, the authors turned to a cultural-clinical psychology framework proposed by Ryder et al. (2011). Introducing the idea of integrating cultural and clinical psychology, they wrote:

We know that ‘culture matters’ in mental health—-but do we know how it matters, or why? Answers may be found in an integration of cultural and clinical psychology. Cultural psychology demands a move beyond description to explanation of group variation. For its part, clinical psychology insists on the importance of individual people, while also extending the range of human variation. Cultural-clinical psychology integrates these approaches, opening up new lines of inquiry” (p. 960).

In other words, this framework not only addresses the limitations of cultural and clinical psychology by integrating the two, but also suggests new ways of looking at a particular psychopathology both theoretically and methodologically. Cultural scripts, local meanings, and influences of social norms and institutional practices are considered, while a focus on psychological mechanisms that account for individual differences is maintained. Culture-mind-brain is thus viewed as a dynamically interrelated system involving multiple levels of analytic sites (i.e., from neural pathways to sociopolitical institutions and global contexts). As a result, research from this paradigm aims to provide “a culturally-framed story about what is observed” (Ryder et al., 2011 p. 960).

Rather than placing excessive emphasis on the peculiarity of symptoms or providing cultural “black box” explanations, examining the egorrhea syndrome from the perspective of cultural-clinical psychology makes it possible to identify issues that are pertinent to a particular culture under consideration. Such efforts may lead to the development of treatments that fit with cultural scripts, local meanings, and sociocultural factors that are specific to that culture.

## Empirical studies on non-clinical samples in japan

The authors have conducted empirical studies on egorrhea using a cognitive-behavioral model (e.g., Sasaki and Tanno, 2003, 2004, 2005a,b,c; Sasaki, 2011). In this section, we will provide a synopsis of three of these studies, which will be followed by a section where we will reinterpret and discuss the results in light of the cultural-clinical psychology perspective. Additionally, it should be noted that the results of these studies have been published in the Japanese language. Nonetheless, reporting the results from these studies in this paper will be beneficial because (a) they will provide the basis for cultural-clinical psychology discussions in the subsequent section and (b) such an effort is in accordance with the recent call for a broader international psychology, which encourages English publications of studies conducted by and on non-Euro Americans (Arnett, 2008).

The three studies that will be discussed have several characteristics. The first feature is that at the start of the research endeavor, we adopted a working definition of egorrhea as *the experience of feeling that one's personal internal information, such as feelings or thoughts, is conveyed to others without the individual saying anything.* Egorrhea patients' presenting concerns are varied, complicated, and often exaggerated. In other words, expressions differ from one patient to another and taking patients' expressions literally makes it impossible to define the phenomenon in a way that can be studied empirically. Thus, we devised this working definition through a careful and extensive review of the existent literature. Whereas the exact definition should evolve as more research illuminating the nature of this phenomenon emerges, articulating a clear working definition helped the researchers maintain a coherent focus throughout the research program. In fact, a pragmatic approach to defining diagnostic categories is consistent with a recommendation by Ryder et al. (2011, in appendix).

Second, our studies employed non-clinical samples of university students in Japan. As mentioned above, due to the resemblance of some egorrhea symptoms to schizophrenia, clinicians and researchers tend to assume that patients who present with characteristics of egorrhea are severely disturbed. It is therefore necessary to understand the extent to which non-clinical populations experience symptoms of egorrhea, rather than just clinical populations. This approach of looking at mild psychopathological symptoms among normal samples is referred to as an analog study. A good example of this method is a study by Rachman and de Silva (1978). Having found that the majority of individuals within a non-clinical population experienced intrusive thoughts, this analog study spurred further clinical research on obsessive-compulsive disorder. Furthermore, studies using non-clinical samples can also shed light on possible adaptive functions of psychological symptoms. Instead of assuming a certain psychological phenomenon is abnormal, such research efforts may make it possible to answer more nuanced questions about the phenomenon, such as when an initially adaptive phenomenon may become dysfunctional.

The third feature of our program of study was a distinction between the prevalence of egorrhea symptoms and the degree to which egorrhea experiences caused distress (Study 2). In clinical samples, patients' feeling that one's internal information revealed to others is generally reported as distressing, resulting in the prevailing perception that the presence of this phenomenon itself is pathological. However, it is possible that an individual may experience a feature of egorrhea but may not be distressed by the experience. If this is the case, the experience of egorrhea may represent an important function of interpersonal communication within a particular cultural context. As such, treatment should focus on an individual's distress rather than the presence of symptoms.

Lastly, these studies employed a cognitive-behavioral model to understand the psychological mechanism of egorrhea syndrome. According to Ellis's ABC model (1977), when an individual encounters an activating event (A: activating event), certain cognitions come to mind (B: belief), which then cause a certain emotional response (C: consequences). This therapeutic modality, which was first developed to treat depression (Beck, 1976), has since been applied to various psychopathologies, such as anxiety disorders (e.g., Clark and Wells, 1995) and schizophrenia (e.g., Beck and Rector, 2000). According to this model, not all people end up feeling negative emotions when encountering the same activating event. Rather, people with particular schemata interpret activating events negatively, which leads to distress. A schema is a mental structure that screens and encodes environmental stimuli in a way that helps the individual to organize the stimuli in a meaningful way (Beck, 1967). Once a schema has developed, it is stable and resists change because counterschematic information gets filtered out or distorted in a way that fits with the schema (e.g., Segal, 1988). Padesky (1994) claimed that schemata linked to negative affective states and maladaptive behavioral patterns are of greatest interest in psychotherapy.

At an early stage in the research program, we applied a cognitive behavioral model and posited the following model of egorrhea as a starting point: *(A: activating event): The individual encounters specific situations linked to egorrhea experiences; (B: belief): The individual interprets the events and believes that his/her personal information is conveyed to others; (C: consequences): The individual feels distressed as a result*. It should be noted that this is a working model, and in order to refine the model, our studies investigated what kinds of situations elicit the experience of egorrhea and what kinds of emotions are experienced in these situations (Study 1) and how prevalent and distressing the experience of egorrhea is (Study 2) as well as what type of cognitive schemata may lead to distress (Study 3). The resultant, revised model will be presented later.

### Study 1: situations eliciting egorrhea symptoms

To understand the nature of egorrhea symptoms, the first study (Sasaki and Tanno, 2003) asked 87 Japanese university students to complete a questionnaire, which consisted of qualitative and quantitative questions. Participants were shown 15 examples of egorrhea experiences, which were developed by Sasaki and Tanno (2003) on the basis of the working definition described earlier in this paper. Participants were asked to describe personal experiences similar to each example in terms of four aspects including: the eliciting situation, other people involved in the situation, thoughts that went through their mind, and emotions they experienced. They were also asked to rate how frequently they experienced each example using a four-point scale. Qualitative data were analyzed using the KJ method (Kawakita, 1967), a sort of content analysis procedure widely used in Japan. The KJ method is an inductive process whereby a conceptual map is created through brainstorming with the use of cards.

The results indicated that 19.8–88.4% of participants experienced each example, with nine out of 15 examples experienced by more than 50 percent of the participants. Additionally, participants reported more than 500 situations in total, which were then grouped into 53 categories. With regards to the emotions experienced by participants, they were mostly negative and potentially distressing. Emotions included embarrassment (25.8%), discomfort (19.2%), impatience (5.6%), chagrin (4.7%), regret (3.7%), and irritation (2.6%). It is noteworthy that embarrassment was the most frequently experienced emotion. This is consistent with Sugawara (1998), who identified that embarrassment is evoked in various situations involving exposure, that is, where others become aware of something that an individual would otherwise keep private. Likewise, with egorrhea symptoms, an individual feels that his or her thoughts or feelings are somehow exposed to others, which seems to be the essence of the experience of egorrhea symptoms.

In contrast, 14.5% of obtained emotional items represented pleasure or some positive emotion, in that participants felt that their thoughts and feelings being conveyed to others was an interesting or beneficial phenomenon. For example, one participant reported, “When I was watching TV, my boyfriend correctly guessed what I was feeling about the show that I was watching. I was pleased that he understands me well.” Another reported, “My mother knew that I was going to trick her. I had no idea how she could possibly know, so I was quite impressed with her.” Common to these statements is a pleasant feeling that one's internal information (i.e., a thought or intention) is conveyed to another person without the individual having to verbalize that information. Importantly, it appears that these experiences made the participants appreciate these relationships, and in addition strengthened their relationship with the other person. In sum, it appears that in some cases egorrhea symptoms elicit positive affect that promotes one's social functioning. That is, the presence of symptoms itself may not necessarily be harmful, and the results of these studies imply that other psychological factors may be responsible for making egorrhea symptoms distressful.

### Study 2: prevalence of egorrhea symptoms and distress level

The purpose of this particular study (Sasaki and Tanno, 2004) was to investigate the prevalence and level of distress caused by egorrhea symptoms. A total of 212 Japanese university students (165 male and 47 female), with a mean age of 18.93 years (*SD* = 1.04), participated in this study. Participants were asked to fill out the Egorrhea Symptoms Scale (ESS, Sasaki and Tanno, 2004).

Developed based on Sasaki and Tanno (2003), this scale focuses primarily on egorrhea symptoms in which the content of internal information conveyed to others is considered negative. This 40-item scale consists of the following five subscales. The “*Disagreeable individual*” subscale (eight items) assesses the extent to which individuals worry that their facial expression unwittingly communicates their sense of awkwardness when talking to someone they dislike or do not know well. The “*Blushing and dismay*” subscale, containing nine items, assesses the symptoms wherein individual worries that others will see that he or she is flustered despite one's efforts to look composed. Seven items in the “*Dirtiness*” subscale assess the extent to which an individual's worries about how others might see him or her as an unhygienic person. The “*Knowing all along*” subscale (eight items) assesses worries that another person, such as one's mother, could know one's whereabouts or actions without having ever been told about them. Finally, the “*Praised*” subscale (eight items) assesses the extent to which an individual worries how others might discern one's sense of pride, for example when praised by a professor in front of others, despite his or her effort to hid this emotion.

The participants were asked to rate how frequently they experience egorrhea and the resulting level of distress on a five-point scale. The prevalence rate for each item was calculated by identifying the percentage of participants who reported having experienced the particular situation at any frequency (i.e., whose response was anything but “no experience”). Likewise, the rate of distress reflected the percentage of participants who reported any level of distress when experiencing the particular situation (i.e., whose response was anything but “not at all distressing”).

The results are shown in Tables 1–5. The prevalence rate ranged from 34 to 86%. For the nine items with a prevalence rate of less than 50%, six items were under the “*dirtiness*” subscale, one item from the “*knowing all along*” subscale, and two items were from the “*praised*” subscale. The remaining 31 items had prevalence rates above 50%. The items with the highest and lowest frequency were “*When I am trying to look calm to hide that I am shocked by my failure in doing something, I feel that my feelings are seen through by those around me*” and “*If it is found out that I have body odor, I feel that I would be considered such a dirty person who doesn't take a bath*,” respectively.

**Table 1:** **Descriptive statistics of “disagreeable individual” subscale**.

**Item**	**Frequency (%)**	**Distress (%)**	**Frequency**		**Distress**	
			**Mean**	***SD***	**Mean**	***SD***
4. When a friend who is not liked so much by many people talks to me, I think s/he might see that I am forcing myself to be nice to and smile at him/her with my face twitching.	57	64	2.0	1.2	2.3	1.2
7. When I am talking to someone who is difficult to deal with, my face twitches, and I feel as if s/he might notice that I do something unnatural.	64	72	2.1	1.1	2.4	1.2
10. When I am speaking with a person I don't like, I suspect that s/he might know that I don't like him/her, because my feelings of dislike could be seen on my face.	66	61	2.1	1.1	2.1	1.2
12. When I speak with someone I don't like, this feeling of mine appears in my eyes and expression without knowing it consciously, so I feel as if s/he might know it.	73	71	2.3	1.1	2.3	1.1
14. When my friend cancels a plan unexpectedly, my face stiffens, although I forgive him/her, I feel as if my disappointment was revealed.	59	59	2.0	1.1	2.1	1.1
32. When I have to talk with someone I am not so acquainted with, I think that s/he might find out from my attitudes and expression that I haven't actually listened to him/her seriously and only pretended to do so.	72	68	2.3	1.1	2.3	1.2
37. When I speak with my friend who I usually think is hard to deal with, I feel as if my feelings might show on my face and s/he might know it.	72	70	2.2	1.1	2.2	1.1
40. When I am speaking with someone I dislike, I feel as if he knew from my expression that I dislike him.	73	64	2.2	1.1	2.2	1.1

**Table 2:** **Descriptive statistics of “blushing and dismay” subscale**.

**Item**	**Frequency (%)**	**Distress (%)**	**Frequency**		**Distress**	
			**Mean**	***SD***	**Mean**	***SD***
2. When I am trying to look calm to hide that I am shocked by my failure in doing something, I feel that my feelings are seen through by those around me.	86	86	2.6	1.1	2.8	1.2
3. When my friends tease me and I blush, I feel as if they knew that I am trying to hide that I am upset and trying to be calm.	83	79	2.7	1.2	2.6	1.2
6. When I blush with my mistakes pointed out in public, I feel as if my embarrassment and shame might appear on my face.	84	82	2.6	1.1	2.8	1.2
11. When I am teased about a relationship with the opposite sex and I blush, I feel as if it might come out that I am upset.	68	61	2.2	1.2	2.1	1.1
15. When someone tells me something mean or asks me to do something I don't like to do, I feel as if my feeling of discomfort might unwittingly show in my look or facial expression.	82	70	2.4	1.1	2.3	1.2
27. When thinking of something I want to stay hidden (e.g., ‘indecent’ thoughts) and catching someone's eyes unexpectedly, I have the feeling my thoughts might be revealed on my face.	67	63	2.2	1.1	2.3	1.2
29. When a passer-by happens to see me stumble while walking alone, I feel that it is apparent that I am upset and trying to keep up appearances.	84	75	2.7	1.1	2.6	1.2
33. When something unpleasant makes me teary, I feel that people around me realize my sad and unpleasant feelings.	71	64	2.3	1.2	2.3	1.2
35. When I make a slip of the tongue out loud but pretend that I don't notice, I feel as if people noticed that I am embarrassed at it.	77	80	2.3	1.1	2.8	1.2

**Table 3:** **Descriptive statistics of “dirtiness” subscale**.

**Item**	**Frequency (%)**	**Distress (%)**	**Frequency**		**Distress**	
			**Mean**	***SD***	**Mean**	***SD***
5. When someone notices my bad breath, I think that s/he would think that I am such a dirty person who doesn't even brush my teeth.	39	64	1.6	0.9	2.6	1.4
16. When I haven't brushed my teeth, I wonder people might notice it by my bad breath.	48	67	1.7	1.0	2.5	1.3
19. When I have to take off the shoes I have worn all day long in order to enter someone's room, I feel that all the people around me might think that I am a dirty person if they notice the vile odor of my feet.	52	68	1.9	1.1	2.5	1.3
22. If it is found out that I have body odor, I feel that I would be considered such a dirty person who doesn't take a bath.	34	59	1.5	0.9	2.5	1.4
25. When someone notices my odor, I feel that he/she might discover that I am a dirty person.	37	62	1.6	1.0	2.5	1.4
28. When I couldn't shampoo my hair the previous day, I feel that, if I have someone notice my hair odor, it would be discovered that I don't have good hygiene.	47	64	1.8	1.0	2.4	1.3
36. When I went to sleep without taking a bath and leave home right after I get up early in the morning, I think that I would be considered a dirty person by people around me if they notice my body odor.	41	66	1.6	0.9	2.5	1.3

**Table 4:** **Descriptive statistics of “knowing all along” subscale**.

**Item**	**Frequency (%)**	**Distress (%)**	**Frequency**		**Distress**	
			**Mean**	***SD***	**Mean**	***SD***
8. Even though I don't tell her anything about it, I wonder how my mother has guessed what I have done that day.	66	59	2.2	1.2	2.1	1.1
17. When my mother finds out that I lied to hide the trick I played, I wonder why she knows it.	58	61	1.9	1.0	2.1	1.1
20. When I tell a lie to my best friend out of vanity, I feel as if he/she were aware that I am showing off, though I am not accused of it.	69	73	2.2	1.0	2.6	1.3
23. When my mother questions me closely about the lie I told, I feel that she might actually find it out.	60	66	2.1	1.1	2.4	1.3
26. When the lie I told to my mother out of vanity is discovered by her, I think it strange why it has been found out by her.	49	51	1.8	0.9	1.9	1.1
30. When I tell a lie to my friend out of desperation but s/he knows the truth, I think it strange why it has been found out by him/her.	55	58	1.8	0.9	2.2	1.2
34. When I cancel a plan with my friend for a false reason -claiming to be ill or fatigued-, I feel that he might know that I lie.	66	73	2.2	1.1	2.7	1.3
39. When I have an inconvenient thing and so I give a random excuse to my best friend, I feel that s/he has already known the fact though s/he says nothing about it.	74	73	2.2	1.0	2.5	1.2

**Table 5:** **Descriptive statistics of “praised” subscale**.

**Item**	**Frequency (%)**	**Distress (%)**	**Frequency**		**Distress**	
			**Mean**	***SD***	**Mean**	***SD***
1. When I am praised in the presence of others for the achievements of my assignment, I feel as if they might see through my proud feelings.	60	60	2.0	1.0	2.1	1.1
9. After getting better marks in tests than my friends, I feel as if my excitement might show on my face.	73	51	2.4	1.1	1.8	1.0
13. When a friend of opposite sex speaks only to me among other friends, I feel as if they might know my proud feelings.	39	42	1.7	1.0	1.7	1.0
18. When my seniors in the same team praise only me in the presence of others, I feel as if the same year friends saw through my happy feelings.	56	47	1.9	1.0	1.7	1.0
21. When I have my paper published anonymously, I feel as if someone might notice through my smirk that I am happy.	47	47	1.8	1.0	1.8	1.0
24. When I am elected as a representative from a few candidates, I feel as if they knew my triumphant feelings.	58	51	1.9	1.0	1.8	1.1
31. When I tell someone about good marks that I received on tests or assignments, I feel as if my proud feelings were apparent to others.	77	64	2.5	1.2	2.2	1.2
38. After receiving better marks than my friend for the university entrance exam, I feel as if he/she knew that I am pleased to have done better.	76	54	2.5	1.2	1.9	1.1

The percentage of participants who reported experiencing distress as a result of experiencing egorrhea symptoms ranged from 42 to 86%. The items with the highest and lowest rates of distress were “*When I am trying to look calm to hide that I am shocked by my failure in doing something, I feel that my feelings are seen through by those around me*” and “*When a friend of opposite sex speaks only to me among other friends, I feel as if they might know my proud feelings*,” respectively.

Table 6 presents descriptive statistics for each subscale. The most important point is that both prevalence and level of distress were high among non-clinical participants. Although it was slightly lower for the “*dirtiness”* subscale (43%), the prevalence rate for the other four categories reached over 60%. The average rate for level of distress was over 50% for all categories. No gender differences were found for either prevalence or level of distress on any of the subscales.

**Table 6:** **Descriptive statistics of the Egorrhea Symptoms Scale**.

**Subscale name**	**Item number**	**Frequency (%)**	**Distress (%)**	**Frequency**		**Distress**	
				**Mean**	***SD***	**Mean**	***SD***
“*disagreeable individual*”	8	67	66	2.2	0.8	2.2	0.9
“*blushing and dismay*”	9	78	73	2.5	0.8	2.5	0.8
“*dirtiness*”	7	43	64	1.7	0.7	2.5	1.1
“*knowing all along*”	8	62	64	2.0	0.8	2.3	0.9
“*praised*”	8	61	52	2.0	0.8	1.9	0.8

While symptoms of egorrhea were previously thought to appear only in clinical populations, this study demonstrates that similar experiences were found to be present and causing distress in non-clinical populations as well. Although this study did not directly compare clinical and non-clinical groups, it appears that the two groups cannot be differentiated based on the presence of symptoms alone. Rather, the differences in two groups may lie, for example, in the level of resiliency and other protective factors that affect an individual's sense of control over egorrhea symptoms. In addition to research that directly compares the prevalence and level of distress between clinical and non-clinical groups, future research should investigate what variables may moderate the level of distress.

### Study 3: relationship between cognitive schemata and distress caused by egorrhea

Prior to Study 3, Sasaki and Tanno (2005a) sought to clarify how egorrhea are experienced and outlined a cognitive model by comparing egorrhea symptoms and two psycho-pathogenic cognitions that have been well studied within CBT—depressive automatic thoughts and obsessive intrusive thoughts. Investigating how similar or dissimilar egorrhea is compared to these two psychopathologies is an important step in creating an explanatory model for egorrhea symptoms. In so doing, Sasaki and Tanno (2005a) selected 10 dimensions most commonly used in multidimensional assessments of psychopathologies (frequency of experience, preoccupation, resistance, intrusiveness, conviction, distress, internal attribution, external attribution, responsibility, and feeling of wrongness) based on Parkinson and Rachman (1981) as well as Clark and de Silva (1985). For each of these dimensions, the Dunnet method of multiple comparisons was used to investigate how egorrhea symptoms differ from intrusive thoughts and automatic thoughts. A total of 54 and 44 university students were administered questionnaires in order to compare egorrhea with intrusive thoughts and automatic thoughts, respectively.

The results showed that the similarity between egorrhea and intrusive thoughts were their low levels of preoccupation and distress, while the similarities between egorrhea symptoms and automatic thoughts were high levels of resistance, conviction, and feelings of wrongness. These results suggest that egorrhea and intrusive thoughts seem to allow for more leeway in thinking about things other than symptoms, and are not particularly distressing in comparison to automatic thoughts. However, individuals with egorrhea symptoms feel that their way of thinking is strange, have stronger convictions, and try not to think about their symptoms in comparison to intrusive thoughts. Although this study showed commonalities between egorrhea and both types of cognitions, particular attention should be paid to the finding that much like intrusive thoughts, egorrhea causes the low levels of distress. As described above, intrusive thoughts are not innately distressing, but can cause distress when a particular schema is triggered.

The above study led to a question which became the focus of Study 3. What kinds of schema are responsible for causing distress when an individual encounters egorrhea related situations? Maintaining a focus on cognitive aspects, Sasaki (2011) explored the relationship between cognitive schemata and distress caused by feelings of self-leakage. A total of 212 university students (164 male, 46 female, and 2 unknown) with a mean age of 19.05 years (*SD* = 0.85) participated in this study. In addition to ESS, participants were asked to fill out the following scales, each of which are purported to assess a particular schema: Praise-Seeking and Rejection-Avoidance Need Scale (Kojima et al., 2003), Other-Offending Cognition Scale (Sasaki and Tanno, 2006), Secrecy Scale (Sasaki and Tanno, 2005b), and Suspicion Scale (Sasaki and Tanno, 2005b). Following data collection, five sets of multiple regression analyses were conducted to examine the relationship between the praise-seeking, rejection-avoidance, other-offending, secrecy, and suspicion schemata with the distress of egorrhea symptoms in each of the five eliciting situations (measured by five subscales of EES), which served as the dependent variable.

The results of the multiple regression analyses were as follows: For the *disagreeable individual* situation, individuals with the other-offending schema (e.g., “*I am offending others by my voice*”), the secrecy schema (e.g., “*Disclosing a secret to others often backfires and results in regretting*”), and the rejection-avoidance schema (e.g., “*As far as possible I avoid discussions where the relationship may turn sour*”) place a negative meaning on egorrhea symptoms and experience distress, which accounted for 26% of the variance. For the *blushing and dismay* situation, individuals with the other-offending schema and the suspicion schema (e.g., “*People are amused by others' secrets when they know it*”) place a negative meaning on egorrhea symptoms and experience distress. These two variables accounted for 31% of the variance. For the *dirtiness* situation, individuals with the praise-seeking schema (e.g., “*I try to charm a stranger, when I first see him/her*”), the suspicion schema and the other-offending schema place a negative meaning on egorrhea symptoms and experience distress, accounting for 14% of the variance. For the *knowing all along* situation, individuals with the secrecy schema, the suspicion schema, and the other-offending schema place a negative meaning on egorrhea symptoms and experience distress, accounting for 20% of the variance. Finally, distress experienced in the “*praised*” situation was predicted by the other-offending and rejection-avoidance schemata, which accounted for 20% of the variance.

In sum, the study revealed that the other-offending schema contributed to all of the egorrhea eliciting situations assessed by the ESS. In other words, people who were distressed by egorrhea symptoms were those who felt they possessed some physical or psychological characteristics that they believed were offensive to others. Based on this study, Sasaki and Tanno proposed a cognitive model of egorrhea syndrome: (a) *Experience of egorrhea symptoms is not uncommon, and it is not necessary pathological nor distressing*, (b) *individuals with specific schemata, particularly the other-offending schema, place a negative meaning on egorrhea symptoms*, (c) *as a result, these individuals experience distress, which may lead to the development of psychopathology.*

## Discussions from cultural-clinical psychology perspective

What kind of cultural story can we tell about egorrhea syndrome based on the above empirical studies? Before plunging into this topic, it is important to acknowledge that these studies are not cross-cultural, as they did not employ comparison groups from another culture. Therefore, we cannot claim cultural differences. Rather, recognizing that empirical research on egorrhea is still in its infancy, we follow Ryder et al. (2011)'s recommendation that inquiry informed by cultural-clinical psychology should “involve using knowledge of the cultural context to propose potential explanations” (p. 974) at an early stage, which could be later tested. Thus, the goal of this section is to discuss the results of the above studies in light of our knowledge on the Japanese cultural context. We hope that our discussions will suggest directions for future cross-cultural research.

### Leakage of self as a normative, adaptive experience

Ryder et al. (2011) argue that “[r]esearch in cultural-clinical psychology should tell us something new about the cultural contexts under study, not just the pathologies” (p. 962). In this sense, the results of the above studies—that non-clinical populations experience features of egorrhea—point to highly complex rules and norms around non-verbal communication in Japanese culture.

As it is well-documented, Japanese culture places a greater value on maintaining group harmony than in expressing one's true self and desires (e.g., Markus and Kitayama, 1991). As such, the cultural value of group harmony demands that individuals be sensitive to how their behaviors impact others around them. One way to achieve this cultural imperative is by adopting the perspective of the outsider phenomenology of the self. According to Cohen et al. (2007), the outsider phenomenology of the self refers to experiencing oneself from the point of view of an outsider or “generalized other” (p, 8) looking at the self, whereas an individual with the insider phenomenology of the self focuses on his or her own private feelings to understand the world. These two phenomenologies coexist; in fact, one's functioning rests on the ability to flexibly switch between the two depending on the demands of various situations.

Yet, a series of empirical studies described in Cohen et al. (2007) indicate that the outsider perspective is more characteristic of East-Asian culture especially in social situations, whereas the insider perspective is more pervasive in Euro-American culture. As such, when taking the outsider perspective of the self, Japanese people tend to pay constant attention to others' behaviors in order to infer how others see them. This finding is consistent with Nisbett et al. (2001) who argue that compared to Westerners who tend to be analytic, East-Asians tend to rely on more holistic cognitive process, that is, attending to context as well as the relationship between the focal object and the field. This type of cognitive process can help the person to take corrective action if he or she detects any negative reactions from other people. This is fairly simple if social cues indicating negative reactions are unambiguous, or if there is any way to verbally confirm what the other person is feeling. However, often such social cues are ambiguous, and the other person is unlikely to express his or her negative feelings verbally, because he or she is also afraid that expressing negative feedback might disturb group harmony (Takai and Ota, 1994). This dynamic forms the basis for *honne* (private, true feelings) and *tatemae* (façade, appearances that are put on public). As a result, one has to read into the other person's *honne* through subtle social cues.

What makes social interactions even more complex is an individual's awareness that other people, abiding by the same social rules, are also trying to read into the person's thoughts and feelings through subtle behaviors such as gaze and blushing. In other words, one is conscious of how his or her behaviors are under scrutiny the same way one pays attention to other's behaviors. Because smooth social interactions rest on one's ability to keep feelings and intentions private, the individual has to censor behaviors that might give away their private feelings. The prevalence of egorrhea experience among non-clinical populations might be explained by such double-tiered (i.e., honne and tatemae), mutually scrutinizing social dynamics.

Readers who are not familiar with Japanese culture might feel that such social dynamics are mentally taxing. It should be noted that other dimensions of the Japanese culture provide safeguards against complex social interactions becoming paralyzing. For example, according to Hofstede's (2001) dimensions of culture, Japan is considered as a tight culture with regards to the uncertainty avoidance dimension. Compared to loose cultures, tight cultures are characterized by the abundance of highly scripted rules and standards for correct behaviors—-for example, how to interact with a stranger. These rules and standards may seem rigid for people from loose cultures where social interactions are more informal. However, these rules and standards in general provide comfort to people in tight cultures, as social conflicts can be avoided as long as the rules are followed. In addition, a minor leakage of *honne* does not automatically result in a social blunder. In situations where one fails to suppress one's internal information, Japanese people often act as if they did not notice anything, as this is considered a respectable or stylish (*iki*) behavior that allows the person to save face.

Furthermore, although embarrassment was by far the most pervasive emotional overtone of experiencing leakage of one's internal information, less frequently but not uncommonly, participants reported positive emotions. As such, Study 1 indicates that this experience may have a possible adaptive function. In Japan where silence is golden, the ideal in close relationships is a state of *ishin denshin*, the literal meaning of which is telepathic communication between minds. *Ishin denshin* signifies a mutual understanding of each other's mind without communicating with words, which helps avoid direct confrontation and interpersonal clashes in a society that is concerns with face-saving (Nishida, 1996).

Of course, *ishin denshin* is not always adaptive, and the notion of in-group vs. out-group seems crucial for understanding the positive function of egorrhea. For those with interdependent selves, individuals' self-esteem rests on their firm sense of belonging to their social groups rather than on their ability to get ahead. Thus, the distinction between in-group and out-group membership is more important for people with an interdependent self-construal than for those with an independent self-construal (Triandis, 2001). The qualitative data from Study 1 suggest that participants reported positive emotions only when others who detected their feelings and thoughts were in-group members (e.g., mother, romantic partner). Thus, it seems that the revealing internal information to in-group members helps Japanese people to affirm their sense of belonging.

Based on these possible cultural explanations, what questions for future research can be generated? First, it would be interesting to examine whether egorrhea experiences are observed among clinical and non-clinical Euro-American samples. If so, to what degree do Euro-Americans feel distressed by or attach a positive meaning to the egorrhea experience? Further, an experimental design could be employed to test the effect of in-group vs. out-group membership of the other person on the level of distress caused by egorrhea.

### Etiology of egorrhea as psychopathology

If the experience of egorrhea is normative to a certain degree, and even adaptive, then when and how does it become pathological? Clearly, the opening case represents a patient whose egorrhea syndrome was so debilitating that it diminished his school and social functioning. We propose that egorrhea syndrome develops as a result of interactions between cultural influences and individuals factors, such as early childhood experiences or tendencies for certain cognitive biases.

Given that the other-offending schema was common in the five egorreha eliciting situations (Study 3), it is crucial to understand how individuals develop and maintain this schema. According to cognitive behavioral theories, schemata are shaped by early childhood experiences (Beck, 1995) and stored in memory (Davis and Unruh, 1981). As the other-offending schema refers to a belief that one's physical or psychological characteristics offend others, it not difficult to presume that individuals with this schema may have received direct negative feedback from others as a child that their physical or psychological features were offensive. Being the target of bullying, neglect or other forms of victimization may form the basis of this schema.

Additionally, certain aspects of Japanese culture may serve to maintain the other-offending schemata even in the absence of direct negative feedback. It is well-documented that education of children in Japan emphasizes consideration of what others feel and thus changing one's behaviors accordingly (e.g., Azuma, 1994). In this context, making internal attributions for negative social events is adaptive, as it can give an impression that one is considerate and caring toward others. In other words, adopting the other-offending scheme as a self-adaptation strategy might be positively reinforced in the Japanese culture. As a result, children who developed the other-offending schema keep trying to find deficits in themselves as a means to elicit positive feedback from their surroundings, which in turn strengthens this schema.

It is also possible to discuss the etiology of egorrhea syndrome in terms of one's inability to effectively adopt the outsider phenomenology of the self. As previously discussed, the outsider perspective, or the seeing oneself from the viewpoint of other people is better suited to the mores of East-Asian culture than the insider perspective. Taking the insider perspective and dwelling too much on one's own thoughts and feelings, on the other hand, can result in a bias about what other people might be thinking and feeling (Cohen et al., 2007). One such bias is the illusion of transparency, which refers to a tendency for people to overestimate the extent to which others can discern their internal states (Gilovich et al., 1998). According to Cohen and Hoshino-Browne (2005), the illusion of transparency is less prevalent among those with an interdependent self-construal such as the Japanese than those with an independent self-construal. This may be because the Japanese are generally confident in their competence to keep separate their internal thoughts and feelings (*honne*) from what they display on their face (*tatemae*), which is integral to social functioning in Japanese culture. It follows that individuals who suffer from egorrhea symptoms are perhaps not as confident in their competence to keep their *honne* separate from their *tatemae*, and thus experience distress in social situations. In fact, Cohen et al. (2007) argued that “[u]nder the “illusion of transparency,” one believes that others can pick up on one's own emotion and discomfort to a greater extent than they actually can; one's own phenomenology of unease dominates” (p. 32). This description markedly resembles the essence of egorrhea.

Another bias that could result from not being able to effectively adopt the outsider perspective is relational projection. Compared to egocentric projection in which people take their own feelings and project *the same feelings* onto others (e.g., an angry person perceive others as angry), Cohen et al. (2007) explain that relational projection involves taking one's feelings and projecting *the complementary feelings* onto others. For example, shame and contempt are complementary in that “[a] person who feels ashamed thinks that others are looking or would look at her with contempt; conversely, a person who feels contempt for another thinks that this person should feel ashamed of herself” (p. 14). This shame-complement pair is particularity relevant to the understanding of egorrhea, given the pervasiveness of embarrassment in egorrehea eliciting situations (Study 1).

It should be noted that the use of relational projection is adaptive particularly in the Japanese context, because certain feelings do co-occur in social situation, and assessing one's emotion can lead to accurate assessment of what other people are feeling (Cohen et al., 2007). In fact, research indicates that taking the outsider perspective, Asian-Americans are more likely to rely on relational projection, whereas Euro-Americans, taking the insider perspective, are more likely to resort to egocentric projection (Cohen and Gunz, 2002). However, error can occur when a particular emotion is too overpowering. In the case of egorrhea, people who suffer from this syndrome may erroneously project contempt onto others, because their own sense of shame and embarrassment is so salient due to the other-offending scheme. Naturally, this relational projection would in turn reinforce their other-offending schema.

The above propositions regarding the etiology of egorrhea syndrome lead to certain questions for future research. For example, future research should examine relationships between egorrhea and variables such as early experiences of victimization, tendency for illusion of transparency, and relational projection. Cross-culturally, it would be interesting to see whether the other-offending schema is also responsible for causing distress, if egorrhea is expressed at all, within Euro-American populations.

## Limitations and contributions

### Limitations

The studies described in the previous section are not without limitations. First, data for all three studies were collected at a national university, where academically achieving young adults are overrepresented. Thus, the results of the studies may not be generalizable to the Japanese population, which spans a wider age range and includes individuals with varying levels of academic achievement. Second, most of the scales used in the studies were developed by the authors themselves. Although these scales have been used by other researchers in Japan, and satisfactory levels of validity and reliability have been reported (e.g., Sakamoto et al., 2005; Hoshino and Tanno, 2009; Takahashi, 2013), further refinement is needed.

The last limitation underlies our attempt to integrate a cultural-clinical psychology framework. Notably, analyses at the brain level are missing both in the scope of the three empirical studies and in our discussions. Although our discussions primarily focused on cultural scripts and norms around social interactions, it would have been interesting to incorporate institutional factors, such as the school or health care system into the analyses. However, acknowledging that a single research project cannot address all levels of the culture-mind-brain system, Ryder et al. (2011) emphasize the importance of researchers collectively contributing “different pieces of the overall puzzle” (p. 969). Thus, despite the above limitations, we believe that our discussion has made several important contributions which may provide an impetus for future research.

### Contributions to cultural psychology and clinical psychology

This paper contributes to the field of cultural psychology in the following ways. First, by synthesizing a body of knowledge that has been accumulated in Japan and introducing that knowledge to Western readers, the foundation has been laid for future cross-cultural dialogs and collaborative research efforts. Second, as a possible cultural explanation, we demonstrated that egorrhea closely reflects cultural values and social norms that govern individuals' behaviors in Japan. Essentially, we argued that egorrhea is a phenomenon occurring as a result of inferring the influence one's presence has on others, as well as ambiguous social cues manifested in other people's behaviors. This act of inferring is driven by a concern for others and how one is perceived by other people. Third, the high prevalence of egorrhea in non-clinical samples and the positive emotions reported by some participants suggests that the experience of self-leakage has an adaptive function. While motivation to belong to a social group as a means to obtain cooperation and resources is innate (Baumeister and Leary, 1995), interdependence and group harmony are particularly integral in collectivistic cultures like Japan. Anticipating negative consequences of self-leakage may help the individual to avoid social mishaps. Then again, what kind of function is played by the positive anticipation of information leaking out? This question requires investigation. Third, as discussed above, it is meaningful to investigate how the other-offending schema develops. How does the childhood of individuals with this schema differ from individuals without? What kind of cognitive biases contribute to the development and maintenance of this schema?

As for the contribution to clinical psychology, there is as yet no evidence-based psychological treatment for egorrhea symptoms. Using a cognitive-behavioral model, the results of our studies are readily applicable to treatment, and we have delineated the following treatment strategies (e.g., Sasaki, 2011). First, the therapist can normalize the patient's experiences and validates their feelings, as people with this syndrome tend to believe that they are abnormal in some ways and are ashamed of these perceived abnormalities. Second, the therapist can help the patient to identify situations, as well as a cognitive schema responsible for eliciting distress. Third, the therapist can encourage the patient to develop and use strategies that help him or her to cope with egorrhea symptoms and provide psychoeducation on the functions of both cognitive and behavioral coping strategies. During this time, the therapist may gradually prompt a shift from coping strategies that are mainly cognitive to those that are behavioral so that desired change gets crystallized in the patient's behaviors. Lastly, the therapist can address and help the patient to modify schema (i.e., offending-others schema) that leads to distress. Tools such as positive data logs and core belief worksheets (Padesky, 1994) can be used to change the maladaptive schema and help patients to develop alternative, more adaptive schemata. These suggestions are obtained from our empirical studies, which form the basis for further studies on the treatment of egorrhea symptoms.

This paper also illuminates future research foci and potential methodological advancements. First, multidimensional assessment methods can be used to explore psychological factors which contribute to individual patients' experiences of unique and idiosyncratic symptoms. Most of the empirical psychopathological research focuses only on the presence or absence of symptoms. However, the presence of symptoms might not be problematic in and of itself, as shown in the studies of egorrhea. Rather, other aspects such as offending others, excessive conviction, or the degree of disturbance in daily life—-that is, the way of experiencing symptoms—-are more important for comprehending the patient's life. For example, if the multidimensional assessment method is applied to the Social Phobia Scale (SPS; Mattick and Clarke, 1998), which is a globally-used scale for assessing the severity of social anxiety symptoms, it is possible to assess other aspects that are meaningful for understanding the patient's idiosyncratic experience, if the items of the SPS are used with modified instructions.

Second, some diagnostic points can be suggested. As mentioned above, when a patient suffering from an anxiety disorder and presents with delusion-like symptoms, it is difficult to judge whether he or she should be diagnosed with anxiety disorder or schizophrenia. As a result, formulating an appropriate treatment plan becomes challenging. Recently, Bizamcer et al. (2008) discussed how to appropriately prescribe medications to patients suffering from ORS, which is one of the egorrhea symptoms. Moreover, the theme that internal information is conveyed to others evokes Schneider's first-rank symptoms and gives the clinician the impression that patients suffer from severe mental health problems. In the case of egorrhea symptoms, as discussed above, having a strong conviction can work to some extent as a cultural adjustment, and suggests that a kind of delusion may be the extreme manifestation of cultural adjustment and a factor other than the presentation and conviction of symptoms may be significant to intervene. In fact, it is also a possibility that individuals with egorrhea symptoms may actually have schizophrenia, and an appropriate way to perform differential diagnosis is still needed.

### The possibility of integration of cultural and clinical psychology

Cultural psychology and clinical psychology complement each other, and research on egorrhea demonstrates several advantages of integrating cultural and clinical psychology. First, a cultural-clinical psychology perspective provides us with a lens to examine egorrhea from a standpoint where neither cultural nor individual factors are overemphasized. As such, this perspective prevents us from assuming that the phenomenon is truly specific to a particular culture, or that the phenomenon is always experienced as distressing. Examining the culture-mind-brain interaction is especially important to develop a comprehensive theory and effective treatment.

Methodologically, the analog study is a suitable method in integrating cultural psychology and clinical psychology. As shown in studies examining egorrhea, the data from non-clinical samples are useful in understanding whether self-leakage is always harmful. Since this was not the case, future research should focus on other dimensions that make egorrehea experiences distressing. Clinical psychology brings in a particular strength in uncovering these dimensions. Additionally, the data from non-clinical samples reflects how individuals function within a specific culture which adds to the knowledge base in cultural psychology.

Finally, the above studies apply a cognitive-behavioral model to understanding the psychological mechanisms of egorrhea. Because previous literature was mainly theoretical, it has been challenging to accumulate empirical evidence. In contrast, a cognitive-behavioral model divides the experiences of patients into discrete parts, including thoughts, emotions, behaviors, (Kuyken et al., 2009), a conceptualization which renders empirical studies more feasible. Thus, rethinking culture-bound syndromes using cognitive-behavioral conceptualization may have the possible advantage of elucidating the exact similarities and differences with other DSM disorders, and of theorizing in greater detail how culture matters.

### Conflict of interest statement

The authors declare that the research was conducted in the absence of any commercial or financial relationships that could be construed as a potential conflict of interest.
